# The Detection of Dental Pathologies on Periapical Radiographs—Results from a Reliability Study

**DOI:** 10.3390/jcm12062224

**Published:** 2023-03-13

**Authors:** Theresa Meusburger, Annika Wülk, Andreas Kessler, Katrin Heck, Reinhard Hickel, Helena Dujic, Jan Kühnisch

**Affiliations:** Department of Conservative Dentistry and Periodontology, University Hospital, Ludwig-Maximilians-University of Munich, 80336 Munich, Germany

**Keywords:** dental caries diagnosis, reliability, reproducibility, caries, periapical pathology, bone loss, periodontitis

## Abstract

(1) Background: Caries, periapical lesions, periodontal bone loss (PBL), and endo-perio lesions are common dental findings that require an accurate diagnostic assessment to allow appropriate disease management. The purpose of this reliability study was to compare the inter- and intra-rater reliability for the detection of the above-mentioned pathologies on periapical radiographs. (2) Methods: Fourteen dentists (three with more than two years and eleven with less than two years of work experience) participated in a training workshop prior to data acquisition. A total of 150 radiographs were assessed by all raters in two rounds. Cohen’s Kappa (CK) values and a binary logistic regression were calculated. (3) Results: The reliability was found in a moderate and substantial range of agreement: caries (mean inter-rater CK value/first round 0.704/mean inter-rater CK value/second round 0.659/mean intra-rater CK value 0.778), periapical lesions (0.643/0.611/0.768), PBL (0.454/0.482/0.739) and endo-perio lesion (0.702/0.689/0.840). The regression model revealed a significant influence of the clinical experience, and furthermore, periapical pathologies and PBL were identified less reliably in comparison to caries and endo-perio lesions. (4) Conclusions: The dentist’s ability to detect the chosen pathologies was linked with significant differences. Periapical lesions and PBL were identified less reliably than caries and endo-perio lesions.

## 1. Introduction

Dental caries and periodontitis affect the majority of the world’s population, and the available epidemiological data demonstrated the widespread prevalence of the diseases [1,2,3,4]. Periapical periodontitis is also among frequent dental findings, as indicated by the fact that half of the global population has at least one tooth with a periapical inflammation [5]. Therefore, an accurate detection and assessment of the mentioned dental pathologies is essential to indicate appropriate disease management [6]. Aiming at reliably detecting and evaluating the existing range of dental pathologies, dental radiography seems to be the adjunct method of choice following the clinical examination [3,7,8].

When analysing the available literature regarding inter- and intra-rater reliability among dental professionals, it appeared that several author groups investigated this issue [9,10,11,12,13,14,15,16,17,18,19,20,21,22,23,24,25,26,27,28,29,30]. Interestingly, the overwhelming majority of scientific reports studied most commonly applied radiographic projection techniques for each pathology and their diagnostic accuracy without considering the inter- and intra-rater reliability. For example, bitewing radiographs are the method of choice for caries detection [6,9,10,11,12,13], periapical and panoramic radiographs for detecting and classifying periapical lesions or PBL [14,15,16,17,18,19,20,21,22,23,24,25,26,27]. In contrast, little is known about the inter- and intra-rater reliability for detecting caries [28] and periapical lesions [14,15,16] on periapical radiographs which are also among the routines in daily dental practice on this image type. In addition, there is limited data available on how reliable dentists detect PBL and endo-perio lesions on periapical radiographs. Furthermore, as known to us, no investigation has analysed the inter- and intra-rater reliability of the previously mentioned dental pathologies on periapical radiographs in one trial to achieve comparable data. Independently from this, it needs to be pointed out that each radiograph has to be viewed for prevalent or rare findings aiming at providing a full assessment of the captured radiographic anatomy. When considering that caries, periapical inflammation, PBL and endo-perio lesions can be easily detected on periapical radiographs, it further justifies the scientific investigation.

Therefore, this diagnostic study aimed to assess the reliability of detecting caries, periapical lesions, PBL and endo-perio lesions on periapical radiographs. The null hypothesis was that there is no difference between the four diagnostic categories and participating dentists.

## 2. Materials and Methods

This reliability study on the diagnosis of pathologies on periapical radiographs was conducted in the context of the research “Detection and diagnosis of dental disorders with artificial intelligence” at the Ludwig-Maximilians University (LMU) of Munich’s Department of Conservative Dentistry and Periodontology. The LMU Medical Faculty’s Ethics Committee gave its approval to this project (project number 20-0798). The current research was presented in accordance with the GRRAS guidelines [31].

### 2.1. Study Participants

A group of 14 dentists with different levels of clinical experience took part in this investigation. Three dentists who were employed at the dental clinic based in Munich had more than two years of work experience; eleven dentists were young professionals and had not worked clinically for longer than two years. Prior to the data collection, the entire study group participated in a 2-day training workshop under the guidance of Prof. Dr. Kühnisch (JK). During the workshop, the study protocol was explained, all pathologies and their detection and classification were described and numerous X-rays were assessed by the study group and each participant individually. After the workshop, the set of periapical radiographs was evaluated twice by all participants. There had to be at least four weeks in between the two assessments to reduce memory bias as best as possible [32]. All dentists were urged to complete the evaluation without assistance and independently.

### 2.2. Set of Periapical Radiographs

A set of completely anonymised periapical radiographs was chosen. All images were recorded at the dental school of the LMU Munich, utilising dental X-ray machines with a 203 mm tube (Heliodent DS, Sirona, Bensheim, Germany) counting an X-ray field restriction (30 × 40 mm) and charge-coupled device (CCD) sensor (Intraoral II, sensor measure 30.7 × 40.7 mm, Sirona, Bensheim, Germany). The exposure time was set at 0.06–0.08 s using a cathode voltage of 60 kV and an amperage of 7 mA. A sensor-holding device (XPP-DS Advanced Sensor Holders for Sirona, Dentsply Rinn, Elgin, IL, USA) was accessible and utilised in the event that it was applicable.

The following diagnostic criteria were chosen for the inclusion of a radiographic image: (1) Periapical radiographs from lower and upper incisors, canines and molars were selected. (2) An image showing the full extent of the main teeth was needed. (3) All X-rays required correct levels relating to exposure, contrast and brightness. Images of teeth with quality defects such as distortions, prominent superimposing effects or others were not selected. (4) At least one radiographic finding—dentin caries, periapical pathology, PBL or endo-perio lesion—should be shown on each image. Finally, 150 periapical radiographs that met the inclusion criteria were identified, and each was assigned a unique identification number.

### 2.3. Diagnostic Standards

**Figure 1:**
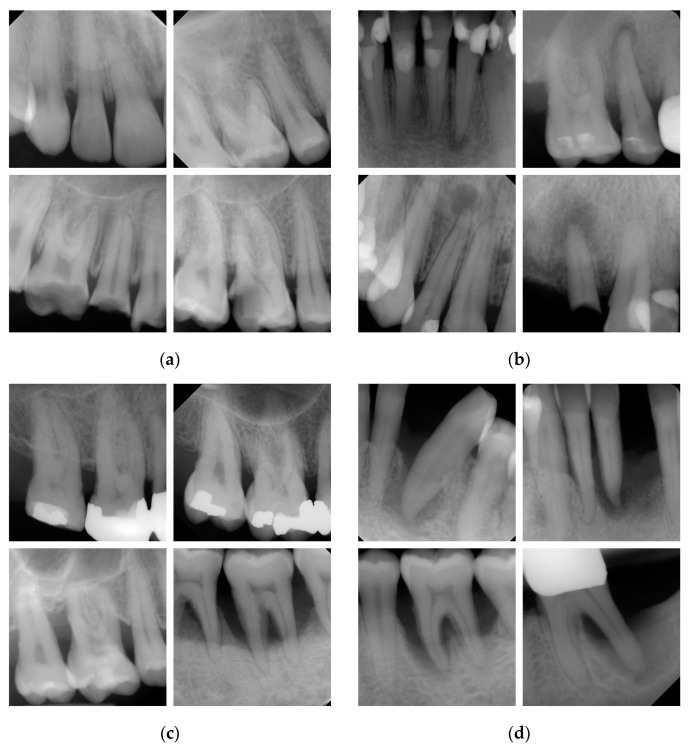
Examples of periapical radiographs for the four criteria: different stages of (**a**) caries, (**b**) periapical lesions (**c**) periodontal bone loss (PBL) and (**d**) endo-perio lesions.

Typical examples for caries (Figure 1a), periapical lesions (Figure 1b), PBL (Figure 1c) and endo-perio lesions (Figure 1d) are shown in Figure 1. All diagnostic standards were defined prior to the data collection as following. The criterion dentin caries was diagnosed according to international accepted recommendations [33,34,35]. When proximal or occlusal caries lesions were registered beyond the enamel-dentin junction or even up to the inner dentin and pulp area, they were registered as dentin caries (Figure 1a). Proximal caries lesions limited to the enamel were not considered. Furthermore, no distinction between different grades of progression was made.

Periapical lesions (Figure 1b) were recorded when a radiolucency around the tooth apex was present. Here, the periapical index by Orstavik et al. [36] classifies periapical pathologies into five different lesion stages. In the present study, periapical inflammation was detected when a radiolucency located in the area around the apex of at least one tooth with twice the width of the periodontal ligament was visible [37,38]. A differentiation between lesion stages was not performed.

To assess the PBL (Figure 1c), the cemento-enamel junction, limbus alveolaris and apex were considered. In detail, the radiographic PBL (cemento-enamel junction—limbus alveolaris) was estimated in relation to the root length (cemento-enamel junction—root apex). If the vertical or horizontal radiographic PBL reached at least the second half of the coronal third of the root length (15–33%), then periodontitis was assumed to be established [39,40,41]. A detailed differentiation under consideration of the exact extent of the radiographic PBL, e.g., into coronal, middle or apical root third [39,40,41] was not made.

Endo-perio lesions (Figure 1d) were detected when the periapical inflammation on a tooth included pulpal and periodontal structures. The classification of endo-perio lesions by Simon et al. [42] served as a reference for this project. Essential radiographic findings included the existence of an inflammation (or radiolucency) that involves the periodontal ligament from the periapical region to the sulcus gingivae.

### 2.4. Consensus Decision (Reference Standard)

Following the two screening assessments, the entire working group re-evaluated all radiographs in an additional meeting to reach a consensus agreement on each X-ray. Again, a yes/no decision (0 or 1) was taken for the four selected criteria. Once one participant presented a deviating result, the team members re-evaluated the concerned radiograph and discussed until agreement was achieved. This decision was set as a reference.

### 2.5. Data Management and Statistical Analysis

An Excel spreadsheet (Excel 2019, Microsoft, Redmond, WA, USA) was used to collect the outcome from the 14 dentists, the 2 assessments (N = 2), and the reference standard. Before analysis, the spreadsheet was checked for plausibility. Using Excel and SPSS, descriptive and exploratory data analysis was performed (SPSS Statistics 27, 2020, IBM Corporation, Armonk, NY, USA). Analysing the inter- and intra-rater reliability of all examiners and the reference standard was part of the statistical study. Cohen’s Kappa (CK), a measure of exploratory analysis, was determined. To obtain a total value, the arithmetic mean of these estimates was computed. The following interpretation must be used for CK values occurring between the ranges listed below: 0.0 to 0.2—slight agreement, 0.21 to 0.40—fair agreement, 0.41 to 0.60—moderate agreement, 0.61 to 0.80—substantial agreement, and 0.81 to 1.00—(almost) perfect agreement [43,44,45]. Additionally, a backward elimination model-based binary logistic regression analysis was carried out for the data (correct/incorrect diagnostic choice with regard to the reference standard and independent variable). The analysis considered the diagnostic decision (caries, periapical lesion, PBL, endo-perio lesion, dependent variable), evaluation round (first vs. second course, dependent variable), and dentists’ experience (</> 2 years of dental work experience, dependent variable) as potential confounding variables.

## 3. Results

The following tables contain the detailed inter- and intra-rater reliability CK values for all participating dentists for the identification of dentin caries (Table 1), periapical pathology (Table 2), PBL (Table 3), and endo-perio lesion (Table 4). Table 5 provides a summary of the mean CK values. In particular, the lowest CK values were found in the first evaluation round of inter-rater reliability for PBL, which was in a moderate agreement range (0.454), followed by the inter CK for periapical pathology (0.643). The mean CK values for caries (0.704) and endo-perio lesion (0.702) were nearly identical. The mean inter-rater CK values for both rounds were discovered in the same value range (Table 5). All diagnostic categories of intra-rater reliability demonstrated a substantial to excellent agreement (0.739 to 0.840; Table 5). The reliability results were found in moderate to substantial agreement for the selected categories in both evaluation rounds, in relation to the reference standard (Table 5). The lowest values were documented for periapical lesions (0.435), followed by PBL (0.575). Both numbers represent a moderate level of agreement.

A binary logistic regression model was applied to further explore the data set (Table 6). The results demonstrated that the variable “evaluation round” had no significant impact on reliability (aOR 0.99, *p*-value < 0.711). On the contrary, the detection of the investigated dental pathologies was documented with significant odds ratios when setting caries as the reference value in the model. Here, the detection of periapical inflammation (aOR 0.34, *p*-value < 0.001) and PBL (aOR 0.57, *p*-value < 0.001) on the selected X-rays was found to be associated with low reliability. In contrast, endo-perio lesions (aOR 1.54, *p*-value < 0.001) were diagnosed with higher reliability. Furthermore, dentists with longer clinical experience (>2 years) evaluated the set of periapical radiographs more consistently (aOR 1.98, *p*-value < 0.001). 

## 4. Discussion

This diagnostic study evaluated the inter- and intra-rater reliability on periapical radiographs for the detection of caries, periodontitis in terms of PBL, periapical inflammations and endo-perio lesions. Generally, the observed CK values were found to be in moderate to substantial agreement (Table 1, Table 2, Table 3, Table 4 and Table 5). The binary logistic regression model (Table 6) showed significant deviations for the detection of the chosen dental pathologies. Additionally, the rater’s clinical experience had a significant impact in the present investigation. Therefore, the initially formulated hypothesis that there is no difference between the diagnostic categories and participating dentists must be rejected.

When discussing the results in detail, caries data should be considered first. Here, we found substantial inter-rater agreement for the first and second evaluation rounds between all raters (mean CK value of 0.704 and 0.659, Table 5) and for the first and second evaluations when the individual decision was related to the reference standard (mean CK value of 0.748 and 0.724, Table 5). The same order of magnitude was registered for the intra-rater reliability (mean CK of 0.778; Table 5). The comparison of our reliability results to other studies with the same methodology [28] indicated basically similar outcomes on periapical radiographs. However, it should be noted that detailed inter- and intra-rater agreement data cannot be taken from this article [28], which hinders further thorough comparisons. Therefore, diagnostic reliability studies for caries detection on bitewing radiographs should be mentioned. Here, numerous studies were published that mostly registered similar CK values for inter- and intra-rater reliability [12,13,29,30]. This conclusion is also supported by recently published data from two systematic reviews and meta-analysis on occlusal and proximal caries detection [9,10].

The reliability results for periapical pathologies in the present study registered substantial inter-rater agreement for both evaluation rounds between all raters (mean CK value of 0.643 and 0.611, Table 5) and moderate agreement for the first and second evaluations when comparing the individual data to the reference standard (mean CK value of 0.435 and 0.442, Table 5). Substantial agreement was recorded for the intra-rater reliability (mean CK of 0.768; Table 5). Due to the fact that periapical radiographs are the projection technique of choice for detecting periapical lesions, there are few publications available that allow comparisons [14,15,16,17]. A study by Patel et al. [14] aimed at detecting periapical pathologies under different viewing conditions by 50 observers. An interesting methodological detail was the feature of obscure tooth crowns on all periapical radiographs aiming at excluding potential diagnostic bias from the rater’s decision, which was not considered in our investigation. As a result, the author group documented fair inter-rater agreement (CK 0.26) among the participating dental students and dentists [14]. This finding is in line with data published by Saunders et al. [17], who found fair to moderate Kappa values for the detection of periapical lesions. A similar observation was drawn by Sebring et al. [18], who documented the greatest variability of diagnostic decisions among teeth with periapical pathologies, which also resulted in fair to moderate agreement; it should be noted that this study was carried out on panoramic X-rays. In contrast to these less-encouraging findings, Patel and co-workers [15] documented an almost perfect agreement for periapical lesion detection (CK 0.878) for two trained endodontists who assessed 30 periapical radiographs. However, the documented reliability data from this study for the detection of periapical inflammation indicate mostly a moderate level of agreement. With respect to the data from logistic regression analysis (Table 6), it needs to be pointed out that in the present study, the detection of periapical pathologies was found to be less reliable in comparison to all other included variables.

When viewing the data in the category PBL, the inter-rater CK values in the two assessments amounted to 0.454 and 0.482 (Table 5), respectively, which represent moderate agreement between raters. In contrast, the inter-rater reliability data in relation to the reference standard showed a substantial agreement for the first and second evaluation rounds (mean CK value of 0.575 and 0.598, Table 5). Additionally, the intra-rater CK value was found to be substantial (0.739, Table 5). In principle, the same reliability data were also documented for endo-perio lesions (Table 5). Surprisingly, we found no comparable investigation that evaluated the ability of dental professionals to detect PBL or endo-perio lesions on periapical radiographs; this limits the discussion and indicates future research needs. However, several publications have assessed the reliability of PBL detection between different radiographic projection techniques or under inclusion of mainly clinical parameters [22,23,24,25,26,40,46,47,48]. When viewing the data from the logistic regression analysis (Table 6), contrary data were observed for the detection of PBL and endo-perio lesions. While PBL was found to be associated with significantly less-reliable readings (OR 0.57, Table 6), endo-perio lesions were detected with a significantly higher detection probability (OR 1.54, Table 6). This becomes explainable by the simple fact that the latter pathology is representing a major finding on periapical radiographs, which is also easy to identify by less-experienced observers.

This research has strengths and limitations that should be discussed. The comprehensive study design, which included 4 diagnostic criteria, 14 raters, and 150 periapical radiographs, must be highlighted as a strength of a reliable study. The four selected criteria represent common dental pathologies that a dentist needs to be routinely diagnose in daily practice on this type of image. Here, it can be further argued that the simultaneous detection of different pathologies is close to the setting in dental practice, where multiple possibilities on X-rays need to be screened and identified correctly if present. In addition, the radiographs and dental structures were fully screened. This may have especially simplified the detection of periapical inflammations, which is frequently associated with profound caries or extensive restorations. This may explain the better CK values in this investigation compared to Patel et al. [14], where the crowns of the teeth on periapical radiographs were obscured to exclude any detection bias. There are also several limitations that must be mentioned. Under daily dental routines, many different factors can be considered, leading to the correct diagnostic decision on radiographic images [8,14,40]. In this study, however, periapical radiographs were evaluated without having access to the clinical background data and the indications that justified their prescription. This may have negatively influenced especially the reference standard. Interestingly, no significant difference was observed between the first and second evaluation round (Table 6). This finding could be addressed mainly to the extensive training before measuring the reliability. In consequence, learning effects during the study were potentially reduced. Furthermore, all included pathologies were rated by all raters as yes or no decision, which was exclusively attributed to the study extent and the workload for each participant. This simplified recording led most likely to more favourable results in comparison to a study design with multiple scores in each diagnostic category. Besides this, other—probably less frequent—pathologies have to be noted and should be known by the dentists, e.g., resorptions, cysts, trauma-induced findings or radio-opacities/radiolucencies. Such pathologies were not considered in the present study project. A similar report was published recently that analysed dentists’ reliability in identifying restorations on periapical radiographs [32] and, interestingly, the CK values for detecting different restorative procedures were found to be better. This finding indicates that different radiographic findings are linked to varying reliability data. In this context, it has to also be mentioned that the dentist’s clinical experience had a significant influence on the outcome in the present study. Here, experienced dentists detected more reliable pathologies on periapical radiographs which simply underlines the need for under- and post-graduate training. With respect to the imbalance in the group of participating dentists (3 experienced vs. 11 inexperienced dentists) and the varying level of clinical experiences, this finding might potentially be biased and, therefore, should not be overrated. Nevertheless, future teaching or training programs should consequently address categories with less reliable findings, e.g., periapical inflammation and PBL. Another limitation might be the fact that different viewing conditions were used by all participants, which were non-standardized and might potentially cause variations during decision making. Here, different computer displays with varying sizes, brightness and contrast settings may have influenced the visibility and detection ability of the participating dentists. However, this is a well-known influencing factor [14,49] which remained uncontrolled in this investigation.

## 5. Conclusions

It can be concluded that this reliability study for detecting caries, periapical pathologies, PBL and endo-perio lesions on periapical radiographs documented moderate to substantial reliability data in terms of Cohen’s Kappa values. Interestingly, the dentist’s ability to detect the chosen pathologies was linked with significant differences. Periapical lesions and PBL were identified less reliably than caries and endo-perio lesions. Further, more experienced dentists made more reliable decisions.

## Figures and Tables

**Table 1:** Values of inter- and intra-rater reliability for caries detection which was calculated among all dentists (N = 14) and in relation to the reference standard (green coloured boxes). Grey coloured boxes illustrate inter-rater CK values of the first assessment and blue coloured boxes for the second assessment. White fields indicate intra-rater CK values.

Cohen’s Kappa Caries			1st Evaluation Round														
		Ref.Stand.	1 *	2	3	4	5	6	7 *	8	9	10	11	12	13	14 *	Mean
	**Ref.Stand.**	1.000	0.804	0.759	0.808	0.587	0.714	0.794	0.804	0.655	0.748	0.759	0.648	0.716	0.808	0.865	0.748
**2nd evaluation round**	1 *	0.776	0.910	0.649	0.771	0.560	0.679	0.758	0.790	0.667	0.820	0.649	0.506	0.654	0.771	0.697	0.690
	**2**	0.808	0.743	0.704	0.731	0.693	0.692	0.745	0.703	0.637	0.622	1.000	0.669	0.746	0.731	0.732	0.719
	**3**	0.836	0.664	0.808	0.863	0.640	0.712	0.765	0.800	0.678	0.686	0.731	0.625	0.714	1.000	0.755	0.739
	**4**	0.692	0.583	0.745	0.719	0.787	0.680	0.627	0.640	0.653	0.613	0.693	0.680	0.733	0.640	0.667	0.655
	**5**	0.611	0.529	0.605	0.638	0.637	0.848	0.754	0.735	0.669	0.707	0.692	0.582	0.946	0.712	0.716	0.714
	**6**	0.772	0.661	0.771	0.772	0.626	0.607	0.838	0.815	0.665	0.758	0.745	0.534	0.782	0.765	0.715	0.725
	7 *	0.879	0.685	0.715	0.771	0.679	0.577	0.705	0.712	0.754	0.880	0.703	0.532	0.737	0.800	0.725	0.740
	**8**	0.558	0.629	0.611	0.615	0.584	0.492	0.582	0.661	0.790	0.725	0.637	0.589	0.699	0.678	0.686	0.672
	**9**	0.760	0.722	0.784	0.704	0.623	0.598	0.673	0.698	0.669	0.863	0.622	0.481	0.710	0.686	0.697	0.693
	**10**	0.699	0.632	0.779	0.672	0.692	0.552	0.664	0.716	0.586	0.758	0.651	0.669	0.746	0.731	0.732	0.719
	**11**	0.657	0.557	0.579	0.657	0.720	0.529	0.562	0.668	0.526	0.571	0.578	0.636	0.633	0.625	0.698	0.602
	**12**	0.731	0.645	0.757	0.731	0.773	0.783	0.746	0.745	0.595	0.686	0.730	0.655	0.960	0.714	0.744	0.735
	**13**	0.563	0.437	0.617	0.696	0.680	0.566	0.654	0.575	0.512	0.529	0.670	0.588	0.668	0.565	0.755	0.739
	14 *	0.790	0.806	0.758	0.762	0.623	0.634	0.674	0.754	0.729	0.849	0.675	0.569	0.714	0.502	0.766	0.717
	**Mean**	0.724	0.638	0.713	0.708	0.668	0.596	0.669	0.688	0.599	0.682	0.670	0.597	0.710	0.592	0.696	0.778

* Skilled study members with more than two years of experience in dental practice.

**Table 2:** Values of inter- and intra-rater reliability for the detection of periapical lesions which was calculated among all dentists (N = 14) and in relation to the reference standard (green coloured boxes). Grey coloured boxes illustrate inter-rater CK values of the first assessment and blue coloured boxes for the second assessment. White fields indicate intra-rater CK values.

Cohen’s Kappa Periapical Lesion			1st Evaluation Round														
		Ref.Stand.	1 *	2	3	4	5	6	7 *	8	9	10	11	12	13	14 *	Mean
	**Ref.Stand.**	1.000	0.664	0.361	0.354	0.337	0.389	0.473	0.410	0.323	0.375	0.361	0.415	0.389	0.340	0.901	0.435
**2nd evaluation round**	1 *	0.678	0.960	0.477	0.461	0.462	0.515	0.618	0.567	0.411	0.475	0.477	0.474	0.541	0.474	0.676	0.510
	**2**	0.333	0.486	0.792	0.759	0.651	0.780	0.766	0.817	0.668	0.799	1.000	0.617	0.814	0.745	0.321	0.709
	**3**	0.378	0.539	0.732	0.827	0.628	0.750	0.792	0.752	0.648	0.704	0.759	0.503	0.781	0.985	0.332	0.681
	**4**	0.357	0.486	0.659	0.631	0.689	0.678	0.668	0.683	0.571	0.728	0.651	0.554	0.711	0.646	0.293	0.610
	**5**	0.389	0.525	0.817	0.787	0.684	1.000	0.756	0.775	0.698	0.757	0.780	0.611	0.967	0.767	0.345	0.706
	**6**	0.487	0.616	0.720	0.815	0.658	0.739	0.897	0.879	0.597	0.772	0.766	0.631	0.787	0.778	0.470	0.714
	7 *	0.901	0.691	0.316	0.359	0.316	0.345	0.460	0.388	0.637	0.824	0.817	0.646	0.807	0.738	0.388	0.718
	**8**	0.275	0.383	0.782	0.715	0.600	0.700	0.644	0.265	0.761	0.651	0.668	0.603	0.698	0.635	0.307	0.599
	**9**	0.368	0.499	0.780	0.817	0.679	0.802	0.768	0.326	0.730	0.918	0.799	0.629	0.790	0.722	0.355	0.693
	**10**	0.326	0.447	0.703	0.777	0.598	0.694	0.732	0.311	0.643	0.758	0.804	0.617	0.814	0.745	0.321	0.709
	**11**	0.509	0.563	0.540	0.574	0.509	0.531	0.592	0.456	0.492	0.527	0.487	0.765	0.642	0.490	0.390	0.570
	**12**	0.386	0.551	0.820	0.822	0.689	0.904	0.742	0.341	0.706	0.837	0.732	0.505	0.936	0.798	0.345	0.730
	**13**	0.265	0.370	0.651	0.659	0.614	0.573	0.626	0.256	0.699	0.673	0.696	0.474	0.582	0.519	0.317	0.680
	14 *	0.532	0.563	0.667	0.730	0.607	0.686	0.798	0.502	0.594	0.744	0.648	0.483	0.718	0.577	0.502	0.374
	**Mean**	0.442	0.517	0.667	0.689	0.595	0.676	0.685	0.380	0.612	0.688	0.633	0.518	0.688	0.573	0.640	0.768

* Skilled study members with more than two years of experience in dental practice.

**Table 3:** Values of inter- and intra-rater reliability for the detection of PBL which was calculated among all dentists (N = 14) and in relation to the reference standard (green coloured boxes). Grey coloured boxes illustrate inter-rater CK values of the first assessment and blue coloured boxes for the second assessment. White fields indicate intra-rater CK values.

Cohen’s Kappa Periodontal Bone Loss			1st Evaluation Round														
		Ref.Stand.	1 *	2	3	4	5	6	7 *	8	9	10	11	12	13	14 *	Mean
	**Ref.Stand.**	1.000	0.610	0.516	0.762	0.428	0.589	0.534	0.748	0.375	0.588	0.516	0.473	0.601	0.658	0.658	0.575
**2nd evaluation round**	1 *	0.658	0.892	0.636	0.453	0.351	0.695	0.263	0.638	0.218	0.479	0.636	0.409	0.682	0.373	0.376	0.478
	**2**	0.502	0.577	0.811	0.401	0.313	0.684	0.216	0.609	0.193	0.478	1.000	0.377	0.700	0.308	0.313	0.479
	**3**	0.796	0.591	0.536	0.779	0.402	0.466	0.601	0.658	0.422	0.549	0.401	0.439	0.476	0.863	0.586	0.517
	**4**	0.569	0.516	0.402	0.450	0.596	0.323	0.275	0.436	0.267	0.271	0.313	0.376	0.331	0.453	0.381	0.346
	**5**	0.472	0.615	0.568	0.502	0.354	0.854	0.291	0.717	0.233	0.559	0.684	0.445	0.958	0.392	0.371	0.524
	**6**	0.529	0.338	0.233	0.498	0.452	0.212	0.767	0.439	0.368	0.431	0.216	0.266	0.298	0.695	0.517	0.375
	7 *	0.706	0.440	0.361	0.585	0.393	0.366	0.463	0.579	0.366	0.653	0.609	0.406	0.731	0.567	0.489	0.563
	**8**	0.512	0.445	0.288	0.608	0.356	0.410	0.403	0.435	0.575	0.522	0.193	0.276	0.269	0.451	0.267	0.311
	**9**	0.620	0.471	0.443	0.743	0.383	0.436	0.431	0.435	0.595	0.800	0.478	0.322	0.572	0.509	0.403	0.479
	**10**	0.565	0.673	0.755	0.548	0.463	0.704	0.239	0.445	0.378	0.455	0.797	0.377	0.700	0.308	0.313	0.479
	**11**	0.473	0.503	0.338	0.473	0.475	0.364	0.286	0.352	0.343	0.398	0.424	1.000	0.456	0.381	0.436	0.382
	**12**	0.650	0.689	0.664	0.634	0.438	0.729	0.310	0.494	0.463	0.620	0.706	0.424	0.889	0.401	0.380	0.535
	**13**	0.620	0.416	0.366	0.639	0.301	0.324	0.486	0.554	0.406	0.599	0.400	0.319	0.445	0.554	0.550	0.481
	14 *	0.699	0.663	0.696	0.737	0.405	0.595	0.340	0.537	0.487	0.645	0.656	0.443	0.755	0.513	0.455	0.414
	**Mean**	0.598	0.534	0.479	0.580	0.414	0.475	0.361	0.451	0.432	0.512	0.527	0.396	0.567	0.444	0.575	0.739

* Skilled study members with more than two years of experience in dental practice.

**Table 4:** Values of inter- and intra-rater reliability for the detection of endo-perio lesions which was calculated among all dentists (N = 14) and in relation to the reference standard (green coloured boxes). Grey coloured boxes illustrate inter-rater CK values of the first assessment and blue coloured boxes for the second assessment. White fields indicate intra-rater CK values.

Cohen’s Kappa Endo-Perio Lesion			1st Evaluation Round														
		Ref.Stand.	1 *	2	3	4	5	6	7 *	8	9	10	11	12	13	14 *	Mean
	**Ref.Stand.**	1.000	0.846	0.736	0.936	0.681	0.918	0.736	0.793	0.589	0.752	0.736	0.397	0.900	0.936	0.968	0.780
**2nd evaluation round**	1 *	0.812	0.963	0.767	0.846	0.706	0.824	0.845	0.868	0.564	0.824	0.767	0.379	0.873	0.846	0.815	0.763
	**2**	0.796	0.760	0.904	0.771	0.650	0.709	0.837	0.902	0.667	0.897	1.000	0.203	0.756	0.771	0.707	0.741
	**3**	0.887	0.827	0.812	0.951	0.645	0.852	0.771	0.828	0.553	0.752	0.771	0.397	0.833	1.000	0.905	0.763
	**4**	0.762	0.797	0.781	0.743	0.767	0.652	0.691	0.772	0.473	0.750	0.650	0.213	0.697	0.645	0.653	0.631
	**5**	0.830	0.723	0.708	0.777	0.671	0.913	0.746	0.770	0.631	0.762	0.709	0.431	0.948	0.852	0.887	0.752
	**6**	0.755	0.789	0.886	0.806	0.810	0.658	0.900	0.941	0.625	0.856	0.837	0.294	0.794	0.771	0.707	0.747
	7 *	0.968	0.782	0.776	0.856	0.733	0.800	0.726	0.763	0.579	0.921	0.902	0.261	0.817	0.828	0.763	0.781
	**8**	0.651	0.712	0.772	0.665	0.696	0.696	0.762	0.623	0.692	0.641	0.667	0.223	0.637	0.553	0.563	0.567
	**9**	0.676	0.740	0.842	0.727	0.723	0.604	0.834	0.648	0.792	0.915	0.897	0.259	0.811	0.752	0.722	0.757
	**10**	0.598	0.652	0.758	0.649	0.677	0.596	0.787	0.572	0.744	0.819	0.849	0.203	0.756	0.771	0.707	0.741
	**11**	0.360	0.379	0.241	0.408	0.410	0.368	0.283	0.340	0.283	0.234	0.257	0.627	0.386	0.397	0.376	0.309
	**12**	0.866	0.910	0.821	0.882	0.821	0.785	0.814	0.835	0.703	0.768	0.685	0.386	0.930	0.833	0.868	0.770
	**13**	0.713	0.708	0.728	0.728	0.692	0.620	0.718	0.685	0.681	0.707	0.661	0.273	0.733	0.713	0.905	0.763
	14 *	0.901	0.840	0.859	0.850	0.824	0.718	0.854	0.870	0.709	0.774	0.693	0.284	0.827	0.773	0.870	0.737
	**Mean**	0.755	0.740	0.750	0.748	0.721	0.671	0.748	0.711	0.680	0.709	0.658	0.319	0.767	0.670	0.760	0.840

* Skilled study members with more than two years of experience in dental practice.

**Table 5:** CK means of the inter- and intra-rater reliability for the four dental pathologies.

Variable	Inter-Rater Reliability	Inter-Rater Reliability	Intra-Rater Reliability	Inter-Rater Reliability	Inter-Rater Reliability
	1st Round	2nd Round	-	1st Round/ Reference Stand.	2nd Round/ Reference Stand.
**Caries**	0.704 (0.602–0.740)	0.659 (0.592–0.713)	0.778 (0.565–0.960)	0.748 (0.587–0.865)	0.724 (0.563–0.879)
**Periapical lesion**	0.643 (0.374–0.718)	0.611 (0.380–0.688)	0.768 (0.388–1.000)	0.435 (0.323–0.901	0.442 (0.265–0.901)
**Periodontal bone loss**	0.454 (0.311–0.563)	0.482 (0.361–0.580)	0.739 (0.455–1.000)	0.575 (0.375–0.762)	0.598 (0.361–0.580)
**Endo-perio lesion**	0.702 (0.309–0.781)	0.689 (0.319–0.760)	0.840 (0.627–0.930)	0.780 (0.397–0.968)	0.755 (0.319–0.767)

**Table 6:** Adjusted odds ratio with corresponding 95% CI and *p*-values were calculated according to the logistic regression model using backwards elimination. Bold numbers represent a significant influence.

Variables	Groups	Odds Ratio	95% CI	*p*-Value
**Categories**	Caries	1	-	-
	**Periapical pathology**	**0.34**	**0.30–0.37**	**<0.001**
	**Periodontal bone loss**	**0.57**	**0.51–0.64**	**<0.001**
	**Endo-perio lesion**	**1.54**	**1.34–1.77**	**<0.001**
**Evaluation round**	1st	1	-	-
	2nd	0.99	0.91–1.07	0.711
**Clinical experience**	≤2 years	1	-	-
	**>2 years**	**1.98**	**1.77–2.22**	**<0.001**

## Data Availability

The datasets generated and analysed during the current study are available from the corresponding author upon reasonable request.
